# Folate Deficiency and Folic Acid Supplementation: The Prevention of Neural-Tube Defects and Congenital Heart Defects

**DOI:** 10.3390/nu5114760

**Published:** 2013-11-21

**Authors:** Andrew E. Czeizel, Istvan Dudás, Attila Vereczkey, Ferenc Bánhidy

**Affiliations:** 1 Foundation for the Community Control of Hereditary Diseases, Torokvesz lejto 32, Budapest 1026, Hungary; E-Mail: i.dudasdr@gmail.com; 2 Versys Clinics, Human Reproduction Institute, Madarasz utca 47-49, Budapest 1138, Hungary; E-Mail: attilavereczkey@hotmail.com; 3 Second Department of Obstetrics and Gynecology, School of Medicine, Semmelweis University, Ulloi ut 78/a, Budapest 1082, Hungary; E-Mail: banhidyferenc@hotmail.com

**Keywords:** neural-tube defects, congenital heart defects, folic acid, multivitamins, primary prevention

## Abstract

Diet, particularly vitamin deficiency, is associated with the risk of birth defects. The aim of this review paper is to show the characteristics of common and severe neural-tube defects together with congenital heart defects (CHD) as vitamin deficiencies play a role in their origin. The findings of the Hungarian intervention (randomized double-blind and cohort controlled) trials indicated that periconceptional folic acid (FA)-containing multivitamin supplementation prevented the major proportion (about 90%) of neural-tube defects (NTD) as well as a certain proportion (about 40%) of congenital heart defects. Finally the benefits and drawbacks of three main practical applications of folic acid/multivitamin treatment such as (i) dietary intake; (ii) periconceptional supplementation; and (iii) flour fortification are discussed. The conclusion arrived at is indeed confirmation of Benjamin Franklin’s statement: “An ounce of prevention is better than a pound of care”.

## 1. Introduction

The aim of this review paper is to demonstrate the breakthrough in the primary prevention of neural-tube defects (NTD) and the possible reduction of congenital heart defects by employing folic acid (FA)-containing multivitamin or folic acid during the periconception period, and to present their three applications in medical practice.

## 2. Birth Defects

Birth defects (this term was introduced in the USA) or congenital anomalies (this term is used by the World Health Organization) comprise all morphologic (structural-anatomic), functional and/or biochemical-molecular defects developing from conception until birth—which are present at birth whether detected at that time or not. However, several categories can be differentiated/classified within birth defects:

Genetic disease with early manifestation (e.g., cystic fibrosis)
Congenital abnormalities or malformations (*i.e.*, structural birth defects)
Congenital tumours (e.g., teratomas)
Idiopathic intrauterine growth retardation (*i.e.*, small newborns for gestational age)
Fetal diseases (e.g., fetal varicella disease)
Immunological disease (e.g., Rh maternal-fetus incompatibility)
Mental retardation
Behavioural deviation
Functional defects of sense organs, e.g., blindness, deafness, *etc*.

Neural-tube defects and congenital heart defects are typical congenital abnormalities with very early (prenatal) onset and defect condition, thus in general there is no or only limited chance for complete recovery. Therefore prevention is the only unique medical solution, and fortunately a breakthrough occurred in the prevention of neural-tube defects by periconceptional folic acid (FA) or folic acid-containing multivitamins (MV). (Multivitamins without FA have no similar preventive effect, and are therefore not discussed here.) The main objective of this review is to show the history of this breakthrough and to present the state of art of this topic.

## 3. Vitamin Deficiencies and Birth Defects

Hale [1] in 1932, *i.e.*, 81 years ago demonstrated that a vitamin A-free diet during the early pregnancy of sows resulted in offspring without eyeballs, with oral clefts, accessory ears, malposition of kidneys and defects of hind legs. Hale’s conclusion was “the condition is illustrative of the marked effect that a deficiency may have in the disturbance of the internal factors that control the mechanism of development” [1]. Later Warkany [2], one of the founders of teratology, recognized the importance of purified diets and he established an experimental model to test various vitamin deficiencies and showed that maternal dietary deficiency induced birth defects. Nelson [3] introduced the use of antimetabolites which made possible conversion of long-term nutritional experiments into short-term chemical testing.

First, FA-antagonists were proved to be highly teratogenic in pregnant rats producing multiple congenital abnormalities, neural-tube defects and orofacial clefts. Later the teratogenic effect of the FA-antagonist aminopteroylglutamic acid (aminopterin) was confirmed in humans as well [4,5,6]. In addition the human teratogenic effect of some other FA-antagonist drugs, e.g., trimethoprim has been shown [7,8].

Second, Hibbard [9] reported in 1964 a higher rate of congenital abnormalities (3.0%) in the infants of folate-deficient mothers than in controls (1.6%). Later Hibbard and Smithells [10] showed a relationship between human embryopathy and a deficiency of folate metabolism. Finally Smithells *et al*. [11] focused their studies on neural-tube defects and demonstrated the role of vitamin deficiencies in the origin of neural-tube defects.

## 4. Neural-Tube Defects

Neural-tube defect (NTD) is the most frequent and the most tragic congenital abnormality of the central nervous system.

The brain and spinal cord develop from the neural-tube which is formed by dorsal folding of the neural plate after the 15th postconception day. The fusion of this folding proceeds in the cranial and caudal directions and is normally completed in humans between the 21st–26th postconception day in the cranial pole and between the 23rd–28th day in the caudal pole, respectively. These periods therefore correspond to the critical period of cranial pole defect: anencephaly and caudal pole defect: spina bifida [12,13]. However, it is necessary to consider that at present the so-called gestational age is calculated from the first day of the last menstrual period in clinical practice, therefore it is necessary to add 14 days to the postconception age to calculate the gestational age. Thus the critical period of anencephaly is between the 35th and 40th gestational day (21 postconception day + 14 = 35 and 26 postconception day + 14 = 40), while spina bifida had this period between the 37th and 42nd gestational days (23 postconception day + 14 = 37 and 28 postconception day + 14 = 42) [12].

Anencephaly is the virtual absence of the forebrain and the skull vault. The clinical manifestation of spina bifida is a midline defect of the osseous spine usually affecting the posterior arches. There are four manifestations of spina bifida: (i) spina bifida aperta with open (uncovered) visible exposed spinal cord; (ii) spina bidida cystica with hernial protrusion of the contents of spinal cord, *i.e.*, meningocele or myelomeningocele covered with a thin membrane; (iii) closed spina bifida with meningocele or myelomeningocele but covered by normal or atrophic skin; (iv) spinal dysraphism, a mild manifestation of closed spina bifida (more than one vertebra is affected with widening of the spinal cord covered by skin with telangiectases, hemangiomas, abnormal pigmentation, hyperthrichosis, lipomas, dimples, dermal sinuses or dermoid cysts). Spina bifida has a typical sequence because very frequently there are secondary congenital abnormalities such as hydrocephalus (in 80% of myelomeningocele cases which usually develops during the first postnatal months), clubfoot and dislocation of the hip, incontinence of urine and feces, as part of placid paralysis of spinal cord lesion [13].

On evaluation of NTD cases it is necessary to differentiate the so-called isolated and multiple-syndromic cases [13]. Isolated NTDs are not associated with other congenital abnormalities, while syndromic NTDs have concurrence of one or more other congenital abnormalities in the same person (e.g., NTD + cleft lip + polydactyly). Syndromic NTDs are caused by chromosomal aberrations (e.g., trisomy 13), mutant major genes (e.g., Meckel-Gruber syndrome with autosomal recessive inheritance) and teratogens (e.g., valprote). However, it is worth mentioning that syndromic NTD cases represent only a minor part of all NTD cases (about 10%), thus the isolated manifestation is characteristic for NTD.

Most isolated NTD cases have multifactorial origin, *i.e.*, polygenic predisposition with an interaction by external agents which can trigger or suppress this genetic predisposition. The polygenic predisposition is confirmed by the recurrence risk that is 10-fold higher in the first degree relatives of patients with NTD than the first occurrence of NTDs in a given population. On the other hand, the importance of environmental agents is indicated by the socio-economic dependence of NTDs (the occurrence of NTDs is much lower in the highest class than in the lowest class), their obvious geographical differences (0.21 per 1000 in Bogota, Columbia and 10.5 per 1000 in North China) and last but not least by the preventive effect of FA and MV [13].

## 5. Short History of Primary Prevention of Neural-Tube Defects

As we mentioned previously, the occurrence of NTDs depends on maternal socio-economic status, thus Smithells hypothesized that undernutrition could be the common factor in the origin of NTDs [11]. Therefore his group tested the effect of diet supplemented with a MV containing 0.36 mg FA in the first intervention trial [14]. Women who had had one or more previous infants with NTD were supplemented by this MV during the periconception period while controls were recruited among similar women, *i.e.*, with previous NTD babies who were already pregnant without vitamin supplementation. The results of this intervention study were published separately for the Yorkshire region of the UK [15] and Northern Ireland [16], where they found 91% and 83% reduction in NTD recurrence, respectively.

However, these results were not accepted by some experts due to possible selection bias in their non-randomized controlled trial (RCT). Thus the Medical Research Council (MRC) in the UK [17] organized a multicenter RCT (43% of participants came from Hungary). There were four supplementation groups: FA (4 mg), other vitamins, FA + other vitamins and minerals as control. The MRC Vitamin Study found that a pharmacological dose (4 mg) of folic acid alone can reduce NTD recurrence by 71% (0.8%, 4.3%; 0.29, 0.12–0.71).

The periconceptional MV supplementation was incorporated into the Hungarian Periconception Service (HPS) launched in 1984 [18] as a randomized controlled trial (RCT). Half of the participants were supplied with a micronutrient combination (the so-called “multivitamin”) containing 12 vitamins, among them, FA: 0.8 mg, B12: 4.0 microgram, B6: 2.6 mg and B2: 1.8 mg, four minerals, and three trace elements, while the other half of the participants were supplied randomly with a placebo-like trace element combination. These women used these supplements at least during one month before conception and at least during two months after conception, *i.e.*, in the periconception period.

There were two major questions of the Hungarian RCT following the publications of the above two “recurrence” trials [14,15,16,17]: (I) “Does MV reduce the risk of first occurrence of NTDs?” About 95% of women who have a fetus or an infant with NTD had no previous NTD pregnancies, thus the prevention of the first occurrence of NTDs would be a real public health success; (II) The pharmacological dose (4 mg) of FA used in the MRC Vitamin Study may have some adverse effects, thus it was necessary to evaluate the physiological dose (1 mg or less) of FA. The point is that the efficacy of a MV containing 0.8 mg FA was tested.

NTD did not occur in 2391 offspring of the MV group, while six NTDs were found in 2471 offspring of no-MV group (*p* = 0.01; RR with 95% CI: 0.07, 0.01–0.13). Thus, the Hungarian RCT demonstrated first that a MV containing 0.8 mg FA acid prevented about 90% of the first occurrences of NTDs [19].

For ethical reasons, the Hungarian RCT could not be continued, thus a cohort controlled trial (CCT) was designed to collect more data regarding the preventive effect of this MV for NTDs and mainly for other congenital abnormalities [20]. All participants in the HPS were supplied with the MV used in RCT while women for unsupplemented cohort were recruited at the 14th week of pregnancy without vitamin use from the regional prenatal care clinics and they were matched to each pregnant woman of the supplemented cohort. The protective effect of this MV for the reduction of NTDs was confirmed in these 3056 “pairs” (1 *vs*. 9; OR with 95% CI: 0.11, 0.01–0.91).

However, the occurrence of syndromic NTDs was not reduced due to this MV either in the RCT or in the CCT [21].

After this the results of only one intervention trial was reported, the efficacy of 0.4 mg FA was shown for the prevention of first occurrence of NTDs in a Chinese-US study [22]. There was 79% reduction in the risk of NTDs in areas with high rates of NTDs (6.5 per 1000), while this reduction was 41% in areas with low rates of NTDs (0.8 per 1000).

This historical review shows that theoretically the primary prevention of NTD can be solved by periconceptional MV or FA supplementation.

## 6. The Importance of Dietary Folate and Synthetic Folic Acid

The natural polyglutamate folate was discovered by Lucy Wills 82 years ago in 1931 [23] and she recommended using the term vitamin 11 as a “twin” of vitamin B12. Later the monoglutamate form of this vitamin, as FA was produced [24]. However, folate is an umbrella term that encompasses all substituted/unsubstituted, oxidized/reduced and mono/polyglutamate forms of pteroyl-l-glutamic acid including the synthetic form of FA. The latter is called vitamin B9 in France and vitamin B11 in the Netherlands and Hungary. The differentiation of dietary folate and synthetic FA is useful from the medical aspect because we have to add these quantities on estimation of this vitamin intake.

Humans cannot produce folate, the major dietary sources of folates are fresh and frozen green leafy vegetables, citrus fruits and juices, liver, wheat bread and legumes, such as beans. Thus the requirement of this water-soluble vitamin is supplied partly by the dietary intakes of folates, partly by the use of synthetic FA, and on estimation of the necessary dose we have to combine these two forms of the vitamin. McPartlin *et al*. [25] estimated that the optimal daily intake of folate/FA in the periconception period is about 0.66 mg per day for the prevention of NTDs. Daly *et al*. [26] demonstrated that the lowest risk of having a child with NTD was related to a red blood cell folate concentration of 906 nmol/L or more. However, practically 8–12 weeks are needed to reach this level after the previously recommended 0.4 mg FA supplementation. The use of 0.8 mg FA resulted in the necessary level of red blood cell folate concentrations at 4.2 ± 3.5 weeks [27].

Thus, the recommended intake of folate/FA advised for a woman of childbearing age, who is sexually active, is daily 0.70 mg [28] or 0.80 mg [29]. In our opinion, 1.0 mg of folate/FA daily seems to be optimal for all pre-pregnant and pregnant women, via the consumption of 0.2–0.3 mg folate through diet and via supplementation with 0.7–0.8 mg FA.

Folate is required for cell division and cell maintenance, because it acts as a co-enzyme in the transfer and processing of one-carbon unit and plays an important role in nucleotide (thymidine) synthesis which is essential for the *de novo* construction or repair of DNA. In addition this vitamin is a key factor in the “site-specific” methylation of the cytosine base in DNA, which regulates epigenetic gene expression. The third major function of folate/FA is the re-methylation of plasma homocysteine to methionine.

The origin of NTDs can be explained by interaction of genes and environmental factors (such as dietary deficiency). Several genetic and environmental factors contribute to the origin of NTDs, here only one, the most established, hyperhomocysteinemia is discussed.

After dietary intake of polyglutamate folates and intended supply of synthetic monoglutamate FA, both are absorbed in the cells of the upper small intestine. FA can be absorbed directly while folate is changed into the monoglutamate form by the folate conjugase (gamma-glutamyl hydrolase) enzyme. In the next step these monoglutamates are converted to dihydrofolate and then to tetrahydrofolate (THF) by dihydroreductase enzymes. THF is the parent compound of all biological active forms of this vitamin. THF is converted to 5,10-methylene-THF and 5-methylene-THF (5-MTHF) before transfer into the hepatic portal vein which then leads to the liver and onwards to the systematic blood circulation and all body tissues.

When meat, fish or plant proteins are digested, amino acids are released, among these is methionine, which is converted to homocysteine. Homocysteine is a toxic metabolite; therefore humans neutralize it normally as soon as possible. On the one hand, homocysteine is metabolized via the trans-sulfuration pathway to form cystathionine catalyzed by cystathionine β-synthase and serine hydroxymethyltransferase. Cystathionine β-synthase requires pyridoxal 5′-phosphate, *i.e.*, vitamin B6 as a cofactor. On the other hand, remethylation of homocysteine to methionine is catalyzed by methionine-synthase. This enzyme requires vitamin B12 as a cofactor and 5-MTHF as the methyl donor. The latter explains the importance of folate-FA deficiency in the origin of NTD.

The most important cause of hyperhomocysteinemia and/or lack of methionine is the polymorphism of MTHF-reductase (MTHFR) gene [30]. MTHFR 677C>T mutation has been identified and it results in a thermolabile variant (Ala222Val) of the MTHFR enzyme with 40% reduction in its activity of the CT (heterozygote) and with very low (about 70% reduction) activity of the TT (homozygote) forms [31]*.* Vitamin B2, *i.e.*, riboflavin is a cofactor of MTHRF enzyme. The enzyme variants due to its mutant gene-pairs cannot effectively catalyze the pathway of 5,10-methylene-THF to 5-MTHF, *i.e.*, the methyl donor for methionine-synthase*.* About 10%–12% of the European population is homozygous (TT) while about 40% are heterozygotes (CT) for this polymorphism of MTHFR gene-pairs. The frequency of TT and CT genotypes is 11.1% and 45.2%, respectively, in the Hungarian population [32].

The lower activity of MTHFR enzyme reduces the production of 5-MTHF and increases the plasma homocysteine level which causes a delay in the closure of the neural-tube, thus indirectly NTD. If the mother is homozygote for this mutation, the risk of NTDs is 2-fold, if both the mother and fetus are homozygotes, the risk of NTDs is increased 6–7 fold. Heterozygotes have a slight increase in risk of NTDs. On the other hand reduced MTHFR activity may also limit the amount of *S*-adenosylmethionine available for critical methylation reactions, impairing the proliferation of cells at the site of neural-tube closure [13].

In recent years, a nature-identical folate of 5-MTHF known as 6*S*-5-MTHF has been synthesized as a calcium salt and marketed as Metafolin^®^ [33].

However, hyperhomocysteinemia may be associated only with a large part of NTDs but this is far from the total spectrum of NTD. Many possible other etiological pathways have been reported [34,35,36,37].

## 7. Congenital Heart Defects

However, there was an unexpected finding of the Hungarian RCT and it was the main reason to organize the previously mentioned CCT. There was a significant reduction of congenital heart defects (CHD) after MV supplementation in the RCT (RR with 95% CI: 0.42, 0.19–0.98) [19,38] and similar reduction was found in CCT (OR with 95% CI: 0.60, 0.38–0.96) [20]. The combination of the results of these two intervention trials suggested a 43% reduction in the risk for CHD (OR 95% CI: 0.57, 0.39–0.85). However, CHD include heterogeneous manifestations and origins of different entities of CHD, the most obvious reduction was found in ventricular septal defects and conotruncal defects.

The population-based observational Atlanta studies [39,40,41] showed that the use of periconceptional MVs reduced the risk of CHD by 24% (OR with 95% CI: 0.76, 0.60–0.97). When evaluating the association with specific types of CHD, these data also suggested that the association was strongest with ventricular septal defect and some conotruncal defects. The birth prevalence of conotruncal defects was reduced by 50% in the Hungarian intervention trials and Atlanta observational studies.

The data set of the Hungarian Case-Control Surveillance of Congenital Abnormalities [42] helped us to evaluate the effect of high doses of FA (there was only one kind of FA tablet containing 3 mg in Hungary during the study period and in general obstetricians recommended 1 and 2 tablets) during the critical period of CHD (*i.e.*, in the second and third gestational months) [43]. This observational study showed a significant reduction in the birth prevalence rate of CHD (OR with 95% CI: 0.86, 0.77–0.98). A population-based observational Dutch Study [44] evaluated folic acid intake (without mentioning folic acid alone or multivitamins) in early pregnancy, and a significant reduction was found in the rate of CHD (OR with 95% CI: 0.82, 0.68–0.98). The major reduction was observed in septal defects. Finally a significant reduction was reported in the birth prevalence of severe CHD in Quebec, Canada after folic acid fortification of grain products [45].

The meta-analysis of both case-control studies (OR with 95% CI: 0.78, 0.67–0.92) and cohort or RCT (OR with 95% CI: 0.61, 0.40–0.92) showed the preventive effect of MVs in the reduction of CHD [46].

FA antagonist drugs, that inhibit dihydrofolate reductase which is required for DNA synthesis, increased the risk of CHD in the children of pregnant women [6]. After trimethoprim-sulfamethazine (co-trimoxazole) use during the second and/or third gestational month of pregnant women, a higher risk of CHD was found in their children [7,8]. A higher risk of CHD was also observed after the intake of certain sulfonamides [47]. However, the most important argument for the role of FA in the pathogenesis of CHD was that the risk of CHD after the use of FA-antagonists without concomitant use of MVs was 7.7 (95% CI: 2.8–21.7) while this risk was only 1.5 (95% CI: 0.6–3.8) after the parallel use of FA-antagonists and multivitamins [7].

On the other hand an association was also found between the higher plasma homocysteine level due to MTHFR gene polymorphism and the higher risk of CHD [48]. The recent meta-analysis of 29 studies showed that both infant and maternal MTHFR C677T polymorphisms may contribute to the risk of CHD [49]. In addition, the risk of maternal MTHFR C677T for CHD was reduced by periconceptional FA supplementation [50].

Thus the available data support that FA or MVs are essential for normal fetal cardiac development during early embryogenesis and these supplements during the periconception period, or in very early pregnancy, reduce the risk for CHD.

## 8. Practical Use of this Primary Preventive Method on NTD and CHD

There are three main possibilities to use folic acid and folic acid-containing multivitamins for women of childbearing age who are capable of becoming pregnant.

### 8.1. Consumption of Folate- and Other Vitamin-Rich Diets

The preconception period is an appropriate time to change the dietary habit and to improve the lifestyle of prospective parents, particularly mothers due to their compliance as they want to do their best to have a healthy baby. Thus it is an important task to advise all women to have a folate- and other vitamin-rich diet from the preconception time onwards.

The usual daily intake of folate is about 0.16–0.20 mg/day in Hungary [51] and this consumption is not significantly higher in other countries [52]. However, the necessary folate/FA consumption is estimated as 0.66–0.70 mg [25,26], thus it is difficult to imagine about a 3.5-fold increase in folate intake every day in anticipation of conception, which would require the consumption of 500 g raw spinach, 900 g boiled spinach or 900 g raw broccoli [53], *i.e.*, about 15 servings of broccoli on each day. The optimal dose of folate/FA for the prevention of CHD is not known. Furthermore, some part of dietary folate is lost through cooking and processing. Finally, extreme increase in the consumption of extra folate from natural food is relatively ineffective at increasing folate status in the human organism [54].

In conclusion, a diet rich in folate is important in general and in the prevention of NTD and CHD, but folate alone cannot completely neutralize the genetic predisposition for NTD and CHD.

### 8.2. Periconceptional Supplementation

Hard evidence is available to advise all women capable of becoming pregnant to have periconceptional (*i.e.*, 2–3 month before and until 3 months after conception) FA or MV supplementation to reduce the major part of NTD and a certain part of CHD risks.

Thus periconception use of FA or MVs would be a simple and useful approach, nevertheless this opportunity is frequently missed.

(i) The major problem is that about 50% of pregnancies are unplanned in the United States, Hungary and many other industrialized countries. If women have unplanned pregnancies and are not using a supplement routinely, they cannot take advantage of this new primary preventive method during the preconception period. The explanation is clear, at the time of first missed menstrual period about the 15th postconception day, when the unplanned pregnancy is recognized, already there is the onset of the closing of the neural-tube.

There are three public health tasks to help prospective pregnant women to increase their use of periconceptional FA or MVs. The first is a strong and widespread educational campaign to suggest the start of the use of FA or MVs immediately after the discontinuation of oral contraceptive pills or other contraceptive methods when couples decide to have a baby. However, unfortunately these campaigns have had only a limited success in all countries [55,56,57,58].

The second important task is to establish a network of preconception or periconception care within the primary health care [59]. The HPS was launched in 1984 [18] and the Hungarian RCT and CCT—presented previously—were based on the HPS. We prefer to use the term periconception instead of the usual preconception because prenatal care usually begins at about the 7–12th week of pregnancy, but the most sensitive and vulnerable time of fetal development is from the 3rd postconception week until the 8th week, and this period is omitted from medical health service, thus embryos are uncared and, in general, unprotected. The preparation for conception is an appropriate time to stop smoking, stop drinking alcohol, and stop taking narcotics and unnecessary drugs with potential hazards to germ cells and later the fetus In addition it is an optimal time for the launch of preconception folic acid or multivitamin supplementation [60].

Our Hungarian experiences have shown that periconception care is feasible and economical, in addition to also providing an appropriate opportunity for nutritional interventions [60].

The third promising possibility is the combination of the oral contraceptive pill with folate [61]. Recently the US FDA (Food and Drug Administration) has approved a new medicinal product comprising drospirenone and ethinyl estradiol as contraceptive components and levomefolate calcium as the folate component.

(ii) Another problem is connected with the dose of FA. Based upon the Hungarian RCT and some observational studies, the CDC in 1992 recommended that “all women of childbearing age who are capable of becoming pregnant should consume 0.4 mg of FA per day for the purpose of reducing their risk of having a pregnancy affected with spina bifida or other NTD” [62], and this recommendation was subsequently followed by several countries. Daly *et al*. [63] showed that the minimum effective dose of FA is 0.2 mg; however, Daly *et al*. [26] also demonstrated that the lowest risk of having a child with NTD was related to a red blood cell folate concentration of 906 nmol/L or more. However, as mentioned previously this level cannot be reached within 4 weeks after the previously recommended 0.4 mg FA supplementation, practically 8–12 weeks are needed to reach the recommended level of 906 nmol/L. In addition the use of 0.8 mg FA resulted in the necessary red blood cell folate concentration at 4.2 weeks [27]. Thus 0.8 mg FA seems to the best choice [64,65].

(iii) A further dilemma is the choice between FA and MVs. The use of a MV in the Hungarian RCT [19] and CCT [20] and in the study of Smithells’ team [15] showed a higher efficacy (about 90%) in the reduction of NTDs than with the MRC Vitamin Study [17] using a high dose (4.0 mg) of FA (71%) and Chinese-US Study [22] using a low dose (0.4 mg) of FA (41%–79%). The usual argument against the use of other vitamins is that the supplementation group of “other vitamins” in the MRC Vitamin Study [17] did not result in a significant reduction in recurrent NTDs. However, it is worth mentioning that there was a 40% reduction (2.6% *vs*. 4.3%) in the recurrent NTDs near to the level of significance after the supplementation of other vitamins.

Another argument for the use of MVs is that this supplementation is also effective for the reduction of CHD, but there are very limited data concerning a similar preventive effect of FA [66].

Finally hyperhomocysteinemia plays a role in the origin of at least some part of NTDs and CHD. Obviously folate/FA is a key factor in homocysteine metabolism, but vitamin B12, B2 and B6 have also a role in the “detoxication” of homocysteine. The combined effect of these four vitamins in MVs may explain its higher efficacy compared to the effect of FA in the reduction of NDTs and CHD [65]. The names of cobalamin (vitamin B12), pirydoxine (vitamin B6) and riboflavin (vitamin B2) are not well-known among pregnant women; therefore we have used the term “fetal protective vitamins” in the HPS [60].

In conclusion MVs seem to be more effective in the primary prevention of NTDs and CHD, although obviously the use of FA is simpler and cheaper.

### 8.3. Food Fortification

Food fortification seems to be the most practical means of supplementation with folic acid and other vitamins for women with unplanned pregnancies. This public health initiative is comparable to the prevention of goiter by the addition of iodine to salt.

In February 1996, the US Department of Health and Human Services [66] ordered food fortification with FA of all cereal grain products at a level of 0.14 mg/100 g beginning January 1998. This adds only about 0.1 mg FA to the average daily folate diet of women of reproductive age, nevertheless there was a 26% reduction in the total (birth + fetal) prevalence of NTDs [67,68]. Canada also introduced a mandatory flour fortification with FA (0.15 mg/100 g of white flour) in September 1998 and 42% reduction was found in the total prevalence of NTDs [69] and 6% reduction in the birth prevalence of CHD [45]. Later many others countries introduced the mandatory flour fortification with FA, and at present this public health project exists in 76 countries [70]. However, mandatory flour or other food fortifications with FA are not available in European countries.

The mandatory flour fortification would be especially important for the large proportion of women with lower levels of education and income who, in general, have difficulties buying more expensive foods rich in folate and other vitamins or MVs and who have unplanned pregnancies more frequently.

Our major concern regarding this approach is the low dose of FA and the lack of vitamin B12. On the one hand there is well-known dose-effect relation in the preventive effect of FA for NTDs, therefore a high dose of FA (4 mg) was recommended for the reduction of recurrent NTD risk. However, later some experts recommended the use of 5 mg FA for all pregnant women [71,72]. In our opinion 0.8 mg is a good compromise [65]. On the other hand vitamin B12 is an independent risk factor in the origin of NTDs [73], and the combination of FA and vitamin B12 can prevent the so-called “masking effect” of FA in patients with pernicious anemia [74].

However, there are two opposing opinions regarding flour fortification with FA.

Results of studies showed that folate deficiency is a precancerous state, but some studies also suggested the possible carcinogenic effect of high doses of FA [75]. Thus there may be a U-shaped risk of cancer connected with the dose of folic acid [76]. Therefore several experts did not recommend the introduction of flour fortification by FA because it may be associated with a risk in older people with precancerous lesions, e.g., in the colon [77]. The recent meta-analysis of data on 50,000 individuals in the randomized trials did not show a higher risk for overall and site-specific cancer incidence after FA or MV use [78]. Thus, the opposing opinion is based on the positive experience of flour fortification in the USA and Canada, and thus Oakley [79] declared that inertia on FA fortification is a public health malpractice.

## 9. Cost-Benefit and Cost-Effectiveness Analysis

There are two types of economic evaluation of preventive methods which can help public health decisions. The first is cost-benefit analysis that values all outcomes in monetary terms, including death and cases of averted disease. The second type of economic analysis is cost-effectiveness analysis that calculates the ratio of net cost (intervention costs minus medical and other direct costs averted from prevention) to the numbers of health outcomes expressed in natural units (e.g., deaths averted) or with a measure that integrates mortality and morbidity, such as quality-adjusted life years.

The U.S. FA-fortification project was evaluated three times, here only the most recent one is mentioned. Grosse *et al*. [80] estimated the cost-benefit-cost ratio as 40:1 thus the estimated economic benefit in U.S. dollars is 312–425 million annually. The cost savings (net reduction in direct costs) were estimated to be in the range of 145–588 million US dollars per year.

The cost of multivitamins, and particularly folic acid is much lower than prenatal diagnosis of NTD fetuses followed by elective termination of pregnancy. On the other hand it is worth mentioning the cost of medical treatment of live-birth babies with NTD. Anencephaly is lethal defect, but patients with spina bifida need complex and long medical treatment. The yearly cost of their treatment is about 20,000 euro in Hungary not to mention the familial tragedy and the multiple handicapped patients. On the contrary generally seven boxes of folic acid-containing multivitamins are needed for the periconception period, and their cost is about 80 euro in Hungary. The cost of folic acid is much lower, again for seven months about 20 euro.

Thus the prevention of affected newborn infants is not only better than their treatment without a limited chance for complete recovery, but prevention results in a significant benefit also from the economic aspect.

## 10. Conclusions

Recent intervention trials showed that about 90% of common and severe NTDs are preventable by periconceptional (at least one month before conception and at least two months after conception) MVs, while about 70% are preventable by periconceptional FA supplementation. Common CHD with birth prevalence of 0.7–1.0 per 1000 was also partially (about 40%) prevented with MVs. There are three possible uses of this new primary preventive method for the prevention of NTDs and CHD: (i) dietary intake of folate, folate rich diet alone is not enough for the prevention of NTDs and CHD; (ii) periconceptional supplementation of FA or MVs, however, women with unplanned pregnancies cannot utilize this simple and cheap method because it needs the onset of FA/MV use at least 1–3 months before conception; (iii) flour fortification with FA, which is not used in Europe. Thus we need to do our best to follow and utilize the meaning of the English proverb attributed to Benjamin Franklin: “An ounce of prevention is better than a pound of care”.
